# A Multidisciplinary Skull Base Board for Tumour and Non-Tumour Diseases: Initial Experiences

**DOI:** 10.3390/jpm14010082

**Published:** 2024-01-10

**Authors:** Jure Urbančič, Saba Battelino, Roman Bošnjak, Tomislav Felbabić, Nejc Steiner, Matej Vouk, Matej Vrabec, Domen Vozel

**Affiliations:** 1 Department of Otorhinolaryngology, Faculty of Medicine, University of Ljubljana, Vrazov Trg 2, 1000 Ljubljana, Slovenia; 2 Department of Otorhinolaryngology and Cervicofacial Surgery, University Medical Centre Ljubljana, Zaloška 2, 1000 Ljubljana, Slovenia; 3 Department of Neurosurgery, University Medical Centre Ljubljana, Zaloška 2, 1000 Ljubljana, Slovenia; 4 Department of Surgery, Faculty of Medicine, University of Ljubljana, Vrazov Trg 2, 1000 Ljubljana, Slovenia; 5 Department of Radiology, University Medical Centre Ljubljana, Zaloška 2, 1000 Ljubljana, Slovenia; 6 Medilab Diagnostic Imaging, Vodovodna 100, 1000 Ljubljana, Slovenia; 7 Department of Diagnostic and Interventional Radiology, General Hospital Slovenj Gradec, Gosposvetska Cesta 1, 2380 Slovenj Gradec, Slovenia

**Keywords:** skull base neoplasms, carcinoma, remote consultation, paranasal sinuses, ear neoplasms, invasive fungal infections, central nervous system neoplasms, cranial nerve disorders, neuroendoscopy

## Abstract

The skull base is the area where various cancerous and non-cancerous diseases occur and represents the intersection of several medical fields. The key is an integrated treatment by specialists of multiple disciplines. We prospectively analysed patients with a skull base disease between August 2022 and 2023 and presented to the Multidisciplinary Skull Base Board (MDT-SB), which takes place once a month hybridly (in-person and remotely). Thirty-nine patients (median age of 58.2 years) were included, of which twelve (30.8%) had a benign tumour, twelve (30.8%) had a malignant tumour, five had an infection (12.8%), and ten (25.6%) had other diseases. For each patient, at least two otorhinolaryngologists, a neurosurgeon, and a neuroradiologist, as well as an infectious disease specialist, a paediatrician, an oculoplastic surgeon, a maxillofacial surgeon, and a pathologist were involved in 10%, 8%, 8%, 3%, and 3% of cases, respectively. In fifteen patients (38%), the MDT-SB suggested surgical treatment; in fourteen (36%), radiological follow-ups; in five (13%), non-surgical treatments; in two, conservative treatments (5%); in two (5%), surgical and conservative treatments; and in one (3%), a biopsy. Non-cancerous and cancerous diseases of the skull base in adults and children should be presented to the MDT-SB, which consists of at least an otolaryngologist, a neurosurgeon, and a neuroradiologist.

## 1. Introduction

The skull base is an anatomical region that constitutes the bottom and support for the brain, cerebellum, brainstem, and medulla oblongata. It is composed of the frontal, ethmoid, sphenoid, and occipital bones, as well as the temporal bones. Important neurovascular structures (such as the brainstem, internal carotid artery, basilar artery, and all cranial nerves) traverse the skull base or its passages (known as foramina and canals), and they can be affected in the case of disease. Due to the complex anatomy and neurovascular structures, the skull base represents a critical anatomical area [1]. Various acquired or congenital diseases can occur at the skull base, including tumours (e.g., meningioma, olfactory neuroblastoma, and middle ear cancer), infections (e.g., skull base osteomyelitis, cholesteatoma, and invasive fungal infection), non-infectious inflammations (e.g., granulomatosis with polyangiitis), injuries (skull base fracture and iatrogenic injury), and other anatomical abnormalities (e.g., meningoencephalocele) [2].

The skull base serves as the intersection of knowledge for several medical specialties, and the primary care of diseases in this area is typically handled by otolaryngologists, neurosurgeons, maxillofacial surgeons, and neuroradiologists. Specialists from other fields may be involved as needed. There are several subdivisions of skull base surgery; otolaryngologists typically classify it into surgery of the anterior and lateral skull base, depending on the approach side. A subspecialist rhinologist performs surgery of the anterior skull base and this involves intervention from the front or through the nose, paranasal sinuses, and/or face. Surgery of the lateral skull base is performed by a subspecialist otologist and involves intervention from the side of the head or through the temporal bone. Neurosurgeons typically collaborate in both procedures. Surgery of the anterior and lateral skull base addresses diseases in the anterior, middle, and posterior skull base areas.

A multidisciplinary approach is fundamental for the best outcome in treating skull base diseases. This can be coordinated through a Multidisciplinary Skull Base Board (MDT-SB), where all physicians involved in patient care, both surgeons and non-surgeons, participate [3]. It has been found that the patient care approach can differ in more than a tenth of cases after MDT-SB consultation compared to care without MDT-SB presentation [4].

Following the example of experiences in the multidisciplinary management of skull base diseases abroad, the initial purpose was to establish the MDT-SB in our tertiary institution, the largest in our country and among the larger ones in our region. This contribution presents the results of treating patients with skull base diseases to the MDT-SB since its establishment in August 2022. The main goal is to present the experiences gained with a hybrid approach to patient care and identify shortcomings.

## 2. Methods

The prospective cohort clinical study was approved by the National Medical Ethics Committee (No. 0120-498/2020-4, 18 January 2021). A part of the study on diseases of the anterior skull base was registered in the clinical trials registry (www.clinicaltrials.gov (accessed on 8 December 2023, No. NCT05607888)). All patients provided written informed consent to be presented to the MDT-SB.

### 2.1. Patient Recruitment

The study began with recruiting patients for presentation to the MDT-SB between 20 August 2022 and 20 August 2023. The only inclusion criterion for MDT-SB consideration was the disease process in the skull base area. An additional inclusion criterion for the study was the patient’s agreement.

### 2.2. Patient Presentation Method

Patients meeting the inclusion criteria were preliminarily presented to one of the regular members of the MDT-SB (article authors) at the University Medical Centre Ljubljana. One member obtained all necessary patient data for the MDT-SB presentation and entered them into an electronic database on the Microsoft Teams platform (Microsoft Corporation, Redmond, WA, USA), ensuring the protection of sensitive data. Upon entry into the database, other regular and facultative members were informed if needed. A final list of patients for preparation at the MDT-SB presentation was published before the scheduled MDT-SB event, creating a meeting on the same platform and inviting all the participants.

The MDT-SB meeting takes place through online and in-person (Department of Otorhinolaryngology) methods. Each MDT-SB event is recorded, and attendance is documented on the Microsoft Teams platform. The patient is presented by their treating physician, who poses the primary clinical question to the board. This is followed by a detailed review of imaging studies through a neuroradiologist and a professional discussion involving otolaryngologists, neurosurgeons, and other facultative members of the MDT-SB. The consultation concludes with a decision that includes an opinion on the diagnosis or treatment of skull base disease.

The consultation decision is entered into a form on the online platform, and an official report is issued by an otolaryngologist from the Clinic for Otorhinolaryngology and Cervicofacial Surgery, University Medical Centre Ljubljana. If necessary, the patient is referred for additional treatment and prescribed therapy.

All patients with skull base cancer are presented not only to the MDT-SB but also to the board for head and neck cancer (ORL-ONCO board) and, if needed, to the chemotherapy board.

### 2.3. Statistical Analysis

The statistical data analysis was conducted using Microsoft Excel for Mac (version 16, Microsoft Corp., Redmond, WA, USA) and IBM SPSS (version 23, IBM Corp., Armonk, NY, USA). The difference between the groups was considered statistically significant if the probability of rejecting the null hypothesis was greater than 95% (*p* < 0.05).

## 3. Results

### 3.1. Patients

During the analysed twelve-month period, 39 patients (59.0% male, 41% female, an average of three patients per month) were presented to the MDT-SB, with a median age of 58.2 years and an age range of 86.3 years (a minimum age of 2.7 years, a maximum age of 89 years, and an interquartile range of 37.3 years). There was no statistically significant age difference between genders according to the Mann–Whitney U test (*p* = 0.966). Detailed data are presented in Table 1.

### 3.2. Disease Analysis

Of the total, twelve patients (30.8%) presented due to a benign tumour, twelve (30.8%) due to a malignant tumour, five due to infection (12.8%; three due to atypical skull base osteomyelitis, one due to mastoiditis, and one due to chronic sclerosing osteomyelitis), three (7.7%) due to meningoencephalocele, two due to bone metabolism disease (5.1%; fibrous dysplasia), one due to autoimmune inflammation (2.6%; granulomatosis with polyangiitis), one due to vascular change (2.6%; aneurysm of the sphenoidal segment of the internal carotid artery), and in three cases (7.7%), the condition was undefined, as the purpose of the consultation was to decide on the diagnostic approach.

Meningioma, schwannoma, cholesteatoma, and haemangioma represented two cases (16.7%) of benign tumours. Squamous cell carcinoma accounted for eight patients (67%). In contrast, adenoid cystic carcinoma, adenocarcinoma, sinonasal undifferentiated carcinoma with a loss of the SMARC-B1 (SWI/SNF-related, matrix-associated, actin-dependent regulator of chromatin, subfamily b, member 1) gene, and olfactory neuroblastoma each represented one case (8%) among all malignant tumours. In twenty-four patients (69.2%), the disease was accessible with anterior skull base surgery, and in twelve patients (30.8%), it involved the lateral skull base.

### 3.3. Analysis of Involved Specialties

For each patient (100%), at least two otolaryngologists were involved: a rhinologist with anterior skull base surgery (JU and DV) and an otologist with lateral skull base surgery experiences (SB and NS), a neurosurgeon experienced in skull base surgery (RB), and a neuroradiologist (MV and MV). An infectious disease specialist was present for four patients (10%) with infections. For three children (8%), a paediatric haematologist–oncologist was present. For three patients (8%), an oculoplastic surgeon was present. For one, a maxillofacial surgeon (3%) was present, and for one (3%), a pathologist was present due to an unclear diagnosis despite multiple biopsies.

### 3.4. Analysis of Proposed Treatment Methods

The MDT-SB recommended surgical treatment for fifteen patients (38%), radiological monitoring for fourteen (36%), non-surgical treatment for five (13%), conservative treatment for two (5%), surgical and conservative treatment for two (5%), and a biopsy for one (3%).

## 4. Discussion

This article presents the results of patient management following the establishment of the MDT-SB in a tertiary institution, the result of decades of effort and experience from senior authors, the innovation and energy of younger authors, and the dedication of the entire skull base team. In addition to professional qualifications, establishing a skull base team requires the selfless commitment of team members to collaborate on work and patient care, a willingness to compromise, and the treatment of a sufficient number of patients with skull base diseases [5]. Lastly, skull base diseases are managed with piety.

Since the MDT-SB meetings are held in the Department of Otorhinolaryngology and Cervicofacial Surgery, regularly monthly, on every first Thursday of the month at 2 PM (the last hour of the working day), and more frequently when necessary (e.g., when waiting is not possible), 39 patients with various skull base diseases, most commonly cancer and benign tumours, were presented. Non-tumour diseases (including inflammations, infections, and injuries) were been discussed, highlighting one of the advantages of the MDT-SB in our institution. Although multidisciplinary consultations are a common practice in treating skull base diseases, the literature on such approaches is lacking, as far as we know. Ferrari et al. (2021) described the treatment of skull base tumours on a multidisciplinary tumour board, but the discussion was limited to tumours [6].

Because the MDT-SB covers a broader spectrum of pathology, more knowledge from various specialists is required compared with that of head and neck cancer boards (ORL-ONCO board). Specialised understanding of skull base anatomy, diagnostic and therapeutic options, and awareness of the latest research or discoveries supporting and guiding patient care are crucial. Key representatives of the MDT-SB (regular members) in our institution include an otolaryngologist, neurosurgeon, and neuroradiologist, which is comparable to foreign institutions [7] (Table 2). During individual preparation for the consultation, regular members may invite specialists from other fields as needed. In our study, for each patient presented to the MDT-SB, two otolaryngologists (one rhinologist and one otologist), a neurosurgeon, and a neuroradiologist were present every time (100%). Among facultative members, an infectious disease specialist was most frequently present, followed by a paediatrician, ophthalmologist, and maxillofacial surgeon. Based on experiences abroad, it was found that an experienced neuroradiologist’s role in the consultation is crucial, as the radiological extent of the disease often changes after a thorough review of imaging diagnostics. Possible reasons for changes in the radiological extent of the disease or disease type are the neuroradiologist’s better familiarity with the patient’s clinical data during the MDT-SB presentation and preparation for the MDT-SB meeting [8]. 

According to experiences abroad, the collaboration between a neurosurgeon and an otolaryngologist in transnasal endoscopic procedures is crucial in reducing the risk of postoperative complications (e.g., cerebrospinal fluid leak), shortening the operation times due to a more precise understanding of the anatomy of the nose and paranasal sinuses, and providing high-quality postoperative care with regular check-ups by an otolaryngologist, enabling endoscopic dressing of the surgical wound. The learning curve for a neurosurgeon in endoscopic transnasal surgery is more gradual, and treatment outcomes are better when collaborating with an otolaryngologist [9]. The collaboration between an otolaryngologist and a neurosurgeon improves comprehensive patient care, fosters innovation, generates ideas, visualises the surgical field, increases efficiency, develops enthusiasm, and provides immediate second opinions [5].

On the MDT-SB, an oncologist is not a regular member of the board since, in our centre, all head and neck cancer patient cases are discussed on the head and neck cancer boards (ORL-ONCO board). In our experience, the ORL-ONCO board relied on the opinion of the MDT-SB in cases of malignant skull base tumours. One of the main advantages of presenting skull base cancer cases to the MDT-SB is the less limited time for discussing each case, which is not feasible in conventional tumour boards that handle other types of head and neck cancers. Indeed, including a radiation oncologist and medical oncologist in cases of tumour pathology would provide even more quality in treating each case where there is a higher likelihood of an excellent response to radiotherapy and systemic therapy. By including oncologists, we avoid additional patient considerations at additional tumour boards, accelerating patient care and preventing treatment by different physicians who may not be familiar enough with the patient.

Since the MDT-SB is open to all physicians dealing with skull base diseases, we intend to include other specialists as regular members of the MDT-SB, including a nuclear medicine specialist who could improve the diagnosis of certain skull base diseases where nuclear medicine methods play an essential role, especially in skull base osteomyelitis [10] and tumours [11]. It would be reasonable to include an endocrinologist in patients with diseases related to the hypothalamic–pituitary axis. A plastic surgeon should be included when questions arise about more complex defect reconstruction. The MDT-SB does not reflect the exact number of treated skull base diseases in the analysed tertiary institution. Most patients not included in the analysis were most likely treated solely in other departments, which were not committed to initially present patients to the MDT-SB. Due to the organisational structure of our medical centre, there are at least three separate departments (the Department of Otorhinolaryngology and Cervicofacial Surgery, Department of Neurosurgery, and Department of Neuroradiology) regularly involved in the MDT-SB. This separation becomes more fixed due to different hospital information systems, meaning patient data cannot easily be retrieved from one department’s system by another. In addition, including a patient on a skull base board is not mandatory for each department or physician. For that reason, in some instances, inclusion on the MDT-SB depends on a physician’s personal decision (e.g., some patients were treated by a neurosurgeon alone). That is why some pathologies were not included in the analysed period.

It is known that a sufficient influx of these patients is crucial for the quality treatment of skull base diseases [5,7]. In comparison with international data, we believe that a comparable number of patients with skull base diseases were treated in our tertiary institution, ensuring high-quality care. Recent prospective multicentric studies, SINTART-1 [12] and SINTART-2 [13], involving five tertiary institutions, analysed 35 patients with resectable locally advanced cancer and 25 patients with unresectable locally advanced cancer. This averaged to 1.4 patients (SINTART-1) or 1 patient (SINTART-2) annually per individual tertiary institution, which was comparable to our study, where we treated 5 patients with resectable locally advanced sinonasal cancer and 2 with unresectable locally advanced sinonasal cancer [12,13].

The MDT-SB includes the management of the paediatric population in addition to adults. For the best results in treating children with skull base diseases, it is recommended to handle at least one case per month [7], which, according to MDT-SB data, we did not achieve. However, based on unpublished data, the number of treated children exceeded this number, as they were treated outside of the MDT-SB. In the future, disseminating information about the MDT-SB, especially to paediatric and neurosurgical departments, would result in the treatment of more children. Due to the unique nature of treating the paediatric population, it would be necessary to consider the composition of a “paediatric skull base team” in the future, which would cooperate with already established paediatric malignant tumour boards.

Although the implementation of the MDT-SB is technically demanding due to the need to coordinate the work commitments of regular members, a high quality can be achieved through a hybrid approach (i.e., in-person or via online platforms). A hybrid implementation of the MDT-SB also allows doctors, especially trainees, to participate in the educational process. The MDT-SB deals with a concentrated array of different skull base diseases that doctors rarely encounter. Nevertheless, the roles of each regular MDT-SB member should be defined and allocated according to the member’s competencies and experiences as in our centre. Some of these roles are also complementary between members (Table 3).

We have implemented a communication format using Microsoft Teams (Microsoft Corporation, Redmond, WA, USA, MacOS: 1.5.00.21551-1.6.00.22155, Windows: 1.5.00.17656-1.6.00.22378, Web: 1.0.0.2022080828-1.0.0.2023081131, Mobile app (iOS): 4.13.0 (100772022133101)-5.14.2 (100772023143702), Mobile app (Android): 1416/1.0.0.2022344703 (2022344730)-1416/1.0.0.2023143401 (2023143401)) to ensure comprehensive coverage of interested parties, whether they can or cannot attend the multidisciplinary board meetings in person. Many other clinical and research groups have reported a heightened demand for such communication protocols during the COVID-19 pandemic [14,15]. Our primary information-sharing method has been through the software within Microsoft Teams. The integration of this online conferencing tool has significantly enhanced collaboration, improved patient care, and facilitated more extensive training for younger colleagues [16].

Although the MDT-SB implementation strategies have already been described, some noteworthy barriers exist in setting up an MDT. According to Vlastos et al.’s (2021) “principles-barriers-solutions model,” these barriers are time, cost, GDPR issues, the need for advanced diagnostic and treatment services, and appropriate reimbursement and policies. To overcome these barriers, the solutions are face-to-face communication of the potential advantages, emphasis on the importance of the discussed pathology (e.g., skull base), the development of a strong team identity (e.g., bulletin boards, logo, etc.), competent and highly motivated peers (e.g., senior residents or junior specialists), a hybrid option, detailed feedback to referring physicians, the communication of benefits to patients and policymakers, integrated care pathways, “smart” applications (e.g., Microsoft Teams), and dedicated centres (i.e., tertiary referral) [17].

In addition to the abovementioned future prospectives, our team intends to further improve the skull base disease management on the MDT-SB by prospectively analysing steps of the decision-making process (i.e., from a decision to present a patient to the MDT-SB, from the physician’s opinion on the management to the final decision of the MDT-SB). This would detect, e.g., the knowledge and experience gaps in management (i.e., diagnosis, treatment, and follow-up) of skull base disease. Decision-making on the MDT-SB is not individual and, therefore, highly vulnerable to direct external influence as it is similar to the process in the acute setting [18]. The whole environment of the MDT-SB minimises the proposed biased thinking of individual models in terms of incomplete information or even emotional influences, stress levels, and lack of diversity [18,19]. Structuring the process or objectivisation is, therefore, difficult since the only unproblematic control is, again, comparing the outcome of the MDT-SB with guidelines or measuring the adhesion [20].

Human assessment is always prone to inter-rater variability. Suppose we would be able to quantify both components: in that case, the true idiosyncratic and cluster of consensuses using proposed techniques [21] and their comparison across the instances of the MDT-SB might shed light on the performance of various MDT-SB members to the point of detecting grey zones in inter-rater agreement [22].

## 5. Conclusions

The expertise of specialists from various fields dealing with skull base pathology is crucial for managing diseases in this area. The platform for consolidating this knowledge is provided by the Multidisciplinary Skull Base Board, meetings of which can take place live, through online platforms, or in a combined (hybrid) manner. Due to the complexity of treating skull base diseases, responsibly presenting the patient to a Multidisciplinary Skull Base Board in a centre specialising in this type of pathology is essential.

## Figures and Tables

**Table 1:** Data on patients with skull base disease presented at a Multidisciplinary Skull Base Board.

G (Age)	Disease	PreTh	Disease Localisation	Conclusion	Specialists
F (52)	ASBO	CONS+SURG	clivus, sphenoidal sinus, pterygopalatine fossa, lateral nasal wall	CONS	ORL, NSRG, RAD, INF
M (78)	ASBO	none	clivus, apex of the pyramid, geniculate ganglion, parapharyngeal space, cavernous sinus, ICA	CONS + SURG	ORL, NSRG, RAD, INF, PATO
M (49)	MENING	SURG	pterygopalatine, infratemporal fossa, cavernous sinus, sphenoidal sinus, sella turcica, clivus	SURG	ORL, NSRG, RAD
F (65)	CS-SBO	SURG	temporal bone, mastoid	SURG	ORL, NSRG, RAD
F (59)	SCHW	none	jugular foramen	FOLLOW-UP	ORL, NSRG, RAD
F (72)	SCC	none	carotid and parapharyngeal space under the skull base	NON-SRG	ORL, NSRG, RAD
M (63)	SCC	SURG + RT	left cavernous sinus, adjacent temporal lobe	FOLLOW-UP	ORL, NSRG, RAD
M (74)	EC	none	temporooccipital bone, occipital condyle, facial nerve canal, jugular foramen	SURG	ORL, NSRG, RAD
M (33)	ACC	SURG + RT	nose, ethmoid cells, pterygopalatine fossa	FOLLOW-UP	ORL, NSRG, RAD
M (71)	SCC	indCT	nose, ethmoid cells, maxillary sinus, epipharynx, sphenoidal sinus, pterygopalatine fossa, orbital apex, cavernous sinus	NON-SRG	ORL, NSRG, RAD
M (58)	SCC	indST	forehead skin, frontal sinus, orbit, dura of the anterior skull fossa	SURG	ORL, NSRG, RAD
F (18)	FD	SURG	orbit, zygomatic bone, frontal bone, sphenoidal sinus	FOLLOW-UP	ORL, NSRG, RAD
F (89)	HEM	none	sphenoidal sinus	FOLLOW-UP	ORL, NSRG, RAD
F (62)	SCC	none	clivus, pterygopalatine and infratemporal fossae, cavernous sinus, Meckel’s cave, orbital apex, superior orbital fissure, apex of the pyramid	NON-SRG	ORL, NSRG, RAD
F (57)	MEC	none	sphenoidal sinus	SURG	ORL, NSRG, RAD
F (5)	FD	none	frontal bone, sphenoid bone, temporal bone	FOLLOW-UP	ORL, NSRG, RAD
M (22)	MEC	none	external auditory canal, roof of the mastoid	SURG	ORL, NSRG, RAD
M (67)	SCHW	none	pontocerebellar angle	SURG	ORL, NSRG, RAD
M (17)	TU?	none	orbit, optic nerve	FOLLOW-UP	ORL, NSRG, RAD, PED, OPS
M (72)	SCC	SURG	maxillary sinus, infratemporal and pterygopalatine fossae, orbit, ethmoid cells	SURG	ORL, NSRG, RAD
M (71)	SCC	none	clivus, sella, parasellar space, suprasellar, both cavernous sinuses and foramina ovale, pterygoid muscles	NON-SRG	ORL, NSRG, RAD
M (72)	SCC	none	ethmoid cells, frontal sinus, orbit, anterior skull base, eye skin	SURG	ORL, NSRG, RAD
F (79)	MENING	none	roof of the sphenoidal sinus, left parasellar space, optic chiasm, optic nerve	SURG	ORL, NSRG, RAD
F (48)	OST	none	frontal sinus, supraorbital	FOLLOW-UP	ORL, NSRG, RAD
M (56)	CHOL	none	middle ear, external auditory canal, temporomandibular joint, intracranial space	SURG	ORL, NSRG, RAD
M (57)	AN-ICA	none	sphenoidal sinus	FOLLOW-UP	ORL, NSRG, RAD
F (70)	PG	none	middle ear, inner ear, jugular foramen, intracranial space	FOLLOW-UP	ORL, NSRG, RAD
F (60)	HEM	none	temporal bone, mastoid	FOLLOW-UP	ORL, NSRG, RAD
F (3)	TU?	none	cavernous sinus, internal carotid artery, orbital apex, superior orbital fissure	FOLLOW-UP	ORL, NSRG, RAD, PED, OPS
M (43)	GPA	none	middle and posterior skull base, Meckel’s cave, pyramid, parasellar, middle ear	CONS	ORL, NSRG, RAD
M (75)	ASBO	none	orbital apex, pterygopalatine fossa, cavernous sinus	CONS + SURG	ORL, NSRG, RAD
M (58)	AC	indCT	nasal cavities, orbit, olfactory fossa	SURG	ORL, NSRG, RAD, INF
F (30)	DC	SURG	roof of the orbit	FOLLOW-UP	ORL, NSRG, RAD
M (9)	ABC	none	body of the sphenoid bone, sphenoidal sinus, middle clivus	FOLLOW-UP	ORL, NSRG, RAD
M (12)	MASTOID	none	mastoid, roof of the middle ear	SURG	ORL, NSRG, RAD, PED
M (24)	MEC	none	roof of the lateral recess of the sphenoidal sinus	SURG	ORL, NSRG, RAD, INF
F (69)	ONB	none	bilateral ethmoid cells, frontal recess, olfactory fossa, dura of the anterior skull base	SURG	ORL, NSRG, RAD
M (37)	SNUC	none	bilateral all nasal cavities, right internal carotid artery, apex of the orbit, anterior skull base, brain parenchyma	NON-SRG	ORL, NSRG, RAD
M 68	TU?	none	superomedial part of the left orbit	BIOPSY	ORL, NSRG, MAFA, OPS, RAD

G—gender; preTh—prior therapy; ASBO—atypical skull base osteomyelitis; MENING—meningioma; CS-SBO—chronic sclerosing skull base osteomyelitis; SCHW—schwannoma; SCC—squamous cell carcinoma; EC—epidermoid cyst; ACC—adenoid-cystic carcinoma; FD—fibrous dysplasia; HEM—haemangioma; MEC—meningoencephalocele; TU?—tumour of unknown aetiology; OST—osteoma; CHOL—cholesteatoma; AN-ICA—internal carotid artery aneurysm; PG—paraganglioma; GPA—granulomatosis with polyangiitis; AC—adenocarcinoma; DC—dermoid cyst; ABC—aneurysmatic bone cyst; MASTOID—mastoiditis; SURG—surgical treatment; CONS + SURG—conservative (antimicrobial) and surgical treatment; CONS—conservative treatment; SURG + RT—surgical treatment and radiotherapy; indCT—induction chemotherapy; indST—induction systemic therapy (immunotherapy); NON-SRG—non-surgical oncological treatment; ORL—otorhinolaryngologist; NSRG—neurosurgeon; RAD—neuroradiologist; INF—infectious disease specialist; PATO—pathologist; PED—paediatric haemato-oncologist; OPS—oculoplastic surgeon; ONB—olfactory neuroblastoma; SNUC—sinonasal undifferentiated carcinoma (SMARC-B1 deficient); MAFA—maxillofacial surgeon.

**Table 2:** Proposed list of physicians and the purpose of their inclusion in establishing the Multidisciplinary Skull Base Board (MDT-SB).

Specialty	Purpose
REGULAR members	
Otorhinolaryngologist	Diagnosing and treating diseases of the skull base
Neurosurgeon	Diagnosing and treating diseases of the skull base
Neuroradiologist	Diagnosing diseases of the skull base
Nuclear Medicine Specialist *	Diagnosing diseases of the skull base
FACULTATIVE members (this list is not exhaustive)	
Infectiologist	Diagnosing and treating infections of the skull base
Paediatrician	Diagnosing and treating diseases of the skull base in children
Maxillofacial Surgeon	Treating diseases involving the temporomandibular joint and jaws or requiring reconstruction with a bone flap
Plastic Surgeon	Treating conditions requiring complex reconstruction
Oculoplastic Surgeon	Treating conditions requiring complex reconstruction of eye structures and adnexa to preserve vision
Pathologist	Addressing dilemmas in the diagnosis of skull base diseases
Clinical Microbiologist	Addressing dilemmas in the diagnosis of skull base diseases

* Nuclear medicine specialists became regular members of the MDT-SB after completing the research. The oncologist is not yet a regular member of the MDT-SB, as patients with skull base cancer are treated at the head and neck cancer board (ORL-ONCO board) dedicated to the treatment of malignant tumours. The ORL-ONCO board relies upon the opinion of the MDT-SB.

**Table 3:** Roles of Multidisciplinary Skull Base Board members (MDT-SB).

Member’s Position	Member’s Role
Junior rhinologist and anterior skull base surgeon AND/OR (depends on the disease type) Junior otologist and lateral skull base surgeon	Primary contact for the inclusion of a patient with skull base disease in the MDT-SB MDT-SB summons Managing the recording of data for a patient included in the MDT-SB into the database (e.g., on Microsoft Teams) Co-organisation of case presentations (bulletin) Presentation of patients with skull base disease to the MDT-SB Control of the MDT-SB agenda Issuance of the MDT-SB report, listing the names of all participating regular and facultative members Managing the recording of data of MDT-SB conclusions into the database
Senior rhinologist and anterior skull base surgeon AND/OR (depends on the disease type) Senior otologist and lateral skull base surgeon	Professional supervision of junior MDT-SB members Leading communication with supervisory bodies and authorities Ensuring adequate material and non-material resources Mentoring in case presentations (bulletin)
Junior neurosurgeon AND Junior neuroradiologist	Co-organisation of case presentations (bulletin) Managing the recording of data for a patient included in the MDT-SB into the database (e.g., on Microsoft Teams)
Senior neurosurgeon AND Senior neuroradiologist	Professional supervision of junior MDT-SB members Assisting communication with supervisory bodies and authorities Ensuring adequate material and non-material resources Mentoring in case presentations (bulletin)

This list of roles is not exhaustive.

## Data Availability

The raw data supporting the conclusions of this article will be made available by the authors on request.
